# Dalton’s Human Physiology

**Published:** 1861

**Authors:** 


A Treatise on Human Physiology.—Designed for the use of Students and practioners of medicine. By John C. Dalton, Jr., M. D., Professor of Physiology and Microscopic Anatomy in the College of Physicians and Surgeons, New York, etc., etc., etc. Second Edition, revised and enlarged. Two hundred and twenty-two Illustrations. pp. 690.

The rapid strides which Physiology is making, assisted by the microscope, vivisections, animal chemistry, etc., are strongly brought to mind by the advent of such works as this. For the past dozen years, Carpenter and his Continental cotemporaries have held almost undivided sway in this realm of Medical Science; and the text-book of the eminent English Physiologist has, hitherto, fully and worthily filled a prominent position in the libraries, alike of student and practitioner. But America, audacious and aggressive, has again planted her *Excelsior* standard on disputed ground, and this volume, with its clear letter and artistic illustrations,—with its admirable, scholarly arrangement, nervous, terse and lucid style, with its vast and laborious experimentation, as fully sustains the claim to pre-eminence in this speciality, as Prof. Gross' *opus magnus* did in another branch, in giving to the world the "most elaborate and complete work on Surgery, ever published in any country." We believe we fully recognize the value of Draper and Dunglison, Carpenter and Kirke, and Todd and Bowman, and yet we unhesitatingly place Dalton at the head of the list, for qualities already enumerated.

We have spoken in the above as if this were a new work, and so, though a second edition only, it is in many respects, as instance: The introduction of an entire chapter on the *Special Senses*,—in the first edition treated only incidentally; new facts and views in regard to the physiology of the *Cranial Nerves*, with a complete re-arrangement of this chapter; new original experiments relating to the function of the *Cerebellum*, and the conclusions to which they lead; considerations respecting the general properties of *sensation* and *motion*, as resident in the nervous system; a new chapter on *Imbibition and Exhalation*, and the functions of the *Lymphatic system*, including the study of endosmosis and exosmosis, and their mode of action in the animal frame,—the experiments of Dutrochet, Chevreuil, Gosselin, Matteucci and others on this subject; the constitution and circulation of the lymph and chyle, a quantitative estimate of the entire processes of exudation and re-absorption taking place in the animal body—besides additions in various parts to the chapters on Secretion, Excretion, the Circulation, and the functions of the Digestive Apparatus. Finally, we have twenty-two new and original illustrations, of which number, five replace others in the former edition (by far the best illustrated work on the subject, even then,) which were considered imperfect, either in design or execution. In this important feature of illustration, Dalton's work is without a peer, either in adaptedness to the text, simplicity and graphicness of design, or elegance of artistic execution.

Philadelphia: Blanchard & Lea, 1861. Chicago: W. B. Keen.

